# Therapeutic Implications of a Polymethoxylated Flavone, Tangeretin, in the Management of Cancer via Modulation of Different Molecular Pathways

**DOI:** 10.1155/2021/4709818

**Published:** 2021-03-05

**Authors:** El-Shaimaa A. Arafa, Noura T. Shurrab, Manal A. Buabeid

**Affiliations:** ^1^Department of Clinical Sciences, College of Pharmacy and Health Sciences, Ajman University, Ajman, UAE; ^2^Center of Medical and Bio-Allied Health Sciences Research, Ajman University, Ajman, UAE; ^3^Department of Pharmacology and Toxicology, Faculty of Pharmacy, Beni-Suef University, Beni-Suef, Egypt

## Abstract

Chemotherapeutics can induce oxidative stress, inflammation, apoptosis, mitochondrial dysfunction, and abnormalities in neurotransmitter metabolism leading to toxicity. Because there have been no therapeutic strategies developed to target inflammation and oxidative stress, there is a continuing need for new and improved therapy. As a result, there has been increasing interest in complementary and alternative medicine with anticancer potential. Studies have shown that the antioxidant activities and anti-inflammatory effects of citrus fruits are promising natural phytochemicals in the development of new anticancer agents. Tangeretin is a naturally polymethoxylated flavone compound extracted from the citrus peel that has shown significant intestinal absorption and adequate bioavailability, with the added benefit of promoting longevity. In addition, tangeretin is known to exhibit considerable selective toxicity to many types of cancer cell proliferation such as ovarian, brain, blood, and skin cancer. Evidence indicates that tangeretin acts through several mechanisms including growth inhibition, induction of apoptosis, autophagy, antiangiogenesis, and estrogenic-like effects. Furthermore, tangeretin works through mitigating levels of inflammatory mediators in the immune system. Using tangeretin in combination with clinically applied anticancer drugs could be a good strategy for increasing the efficiency of these agents and protecting noncancerous cells from damage caused by chemotherapy. The purpose of this review is to highlight the protective effects of a novel natural product, tangeretin against chemotherapeutic-induced toxicity. The development of chemoprevention strategies can lead to significant health care improvement in cancer survivors. Thus, study outcomes may attract more investigators to conduct tangeretin-related research and find out potentially significant impacts on health care of cancer patients and decreased health problems associated with chemotherapeutics-induced toxicity.

## 1. Introduction

Cancer is the second-leading cause of death globally. More than 14,000,000 new cases are expected and more than 8,000,000 deaths per year, which is translated as about 13% of deaths worldwide [1]. Cancer chemoprevention is pharmacological intervention from a natural, synthetic, biological, or chemical origin that blocks or reverses carcinogenesis. Many forms of cancer are hardly eradicated through cancer chemotherapies, which unfortunately have serious toxicity [2, 3] and unavoidable consequences associated with cancer therapy [4]. Chemoprevention approaches have generated much expectation and interest as adjuvants as have the chemotherapeutic agents in cancer therapy.

Flavonoids are naturally occurring polyphenols that have shown clearly their selective toxicity to cancer cells. Polyphenols inhibit carcinogen-activating enzymes and have various antioxidant properties [5]. Fruits, vegetables, grains, and traditional medicinal herbs are an abundant source of flavonoids [6,7]. Several epidemiologic studies suggested a protective role of flavonoids on certain cancer types, for instance, lung, breast, colon, and prostate [8, 9]. Citrus fruits are an example of chemopreventive and cochemotherapeutic agents containing flavonoids that are associated with cancer treatment [10]. Tangeretin (4′, 5, 6, 7, 8-pentamethoxyflavone) is a natural polymethoxyflavone (PMF) compound, extracted from citrus peel [11] with more than one mechanism of anticancer activity [12]. In the present review, we postulate, from the current evidence on tangeretin use, its potential use as an agent for cancer prevention and/or chemoprevention.

## 2. Biological Functions of Tangeretin

**Figure 1:**
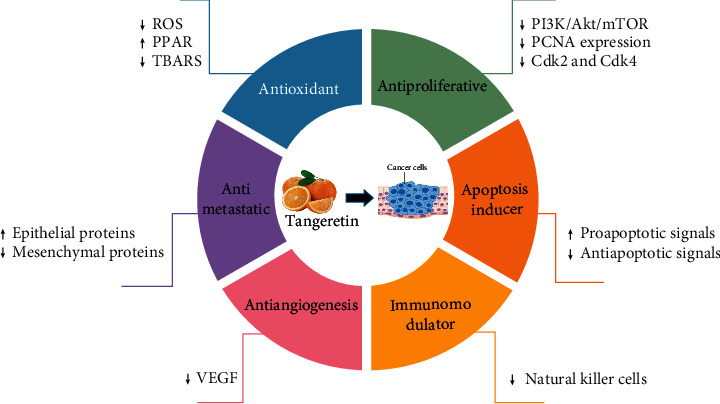
Mechanisms of the anticancer activity of tangeretin. Cdk2, cyclin-dependent kinases 2; Cdk4, cyclin-dependent kinases 4; PCNA, proliferating cell nuclear antigen; PI3K/Akt/mTOR, phosphatidylinositol 3-kinase/protein kinase B/mammalian target of rapamycin; PPAR, peroxisome proliferator-activated receptor; ROS, reactive oxygen species; TBARS, thiobarbituric acid reactive substances; VEGF, vascular endothelial growth factor.

Several studies have shown that tangeretin has significant health-enhancing effects. Tangereting has been reported to reduce blood sugar [13–15], inhibit inflammation [16–22], and oxidation [16, 19]. In addition, it acted as an antiviral [23], prevented heart diseases [24], hepatitis [17, 25], kidney dysfunction [19], and hyperlipidemia [26]. Moreover, the capacity for tangeretin is remarkable in some neurological disorders, including Alzheimer's disease [27], epilepsy, and Parkinson's disease [28]. Tangeretin has further been reported to have an anticancer effect in traditional Chinese medicine [29]. Tangeretin has been observed to inhibit the development and progression of various kinds of cancer cells [29–33], as discussed below: the mechanism of tangeretin is summarized in Figure 1.

### 2.1. Potential Role of Tangeretin as an Antioxidant

Oxidative stress plays a critical role in cancer growth and progression. Evidence indicates that, in the initial phase of cancer, oxidative stress is prevalent [34]. Free radicals generated are known to damage lipids, proteins, and DNA in patients receiving chemotherapeutics. Superoxide dismutase, a key antioxidant enzyme is a major target of oxidative damage. Superoxide dismutase is oxidatively modified by carbonylation. The cysteine residue of the superoxide dismutase is oxidized to cysteic acid. Several lines of evidence implicate lipid peroxidation in the progression of cancer. 4-Hydroxy-2-trans-nonenal (HNE), an aldehydic product of membrane lipid peroxidation, is increased due to oxidative stress. Increased DNA oxidation, especially 8-hydroxy-2′-deoxyguanosine (8-OHdG), is present in the tumor due to oxidative stress. Glutathione (reduced form) levels were depleted in cancer and similarly in the breast cancer model [35]. A tangeretin antioxidant effect was found to balance the defense system. Over accumulation of reactive oxygen species result in oxidative stress, resulting in DNA, RNA, and protein oxidation, as well as lipid peroxidation by indirectly promoting cellular glutathione in renal tissues [36] and HepG2 cancer cells [37]. Since the exact cause of oxidative stress pathophysiology remains controversial, several therapeutic strategies have been tried to prevent or slow progression.

### 2.2. Suppression of Carcinogenesis by Tangeretin

Carcinogenesis is a complicated procedure, including many DNA and non-DNA modifications which ultimately promote the conversion of a normal cell into a cancerous one [38]. The antimutagenic effect of tangeretin on different mutagens such as 2-aminofluorene, benzo[a]pyrene, and nitroquinoline N-oxide was reported using a salmonella/microsome assay. [39]. The antimutagenic effect of tangeretin was further confirmed using the Ames test [40]. Additionally, tangeretin was reported to prevent induced unscheduled DNA synthesis in rat hepatic slices [41]. *In vivo* studies showed the capability of tangeretin to protect against 7, 12-dimethylbenz[a]anthracene (DMBA) induced breast cancer in rats [33, 36].

### 2.3. Effect of Tangeretin on Cell Cycle Regulation

The cell cycle is the process by which cells grow and divide. Regulatory proteins control the cell cycle by either tumor suppression of cell growth or death of damaged cells. Cyclin-dependent kinases (CDK) cyclin complexes are the cell cycle protein machinery controlling cell proliferation under specific stimuli. Cancer growth has been associated with defects in CDK as evidence by an *in vitro* study on COLO 205 human colon cancer. In this study, administration of tangeretin was able to block (G1 phase) by activating the expression of CDK inhibitors p27 and p21 [30]. In another study supporting the anticancer effect of tangeretin on breast cancer cell line (MCF-7), inhibition of cell proliferation was shown to arrest the cell at the G1 phase [42].

### 2.4. Effect on Apoptosis

Cell death, particularly apoptosis, is critical for balanced cell death and growth to maintain body functions [43]. Cancer causes a defect to occur in any point in apoptotic pathways resulting in malignant cells that will not perish [43]. One example is the reduced expression of p53, a tumor suppressor gene, which alters apoptosis and enhanced carcinogenesis. Tangeretin exerts anticancer activity by inhibiting the growth as well as the progression of cancer cells in both in vitro and *in vivo* studies. Results demonstrated that tangeretin possessed selective effectiveness against tumor cell lines [44]. In the studies using a colon carcinoma model [30] and HL-60, human promyelocytic leukemia [45], tangeretin treatment significantly evoked apoptosis by enhancing the expression of p53. Similarly, in rats' breasts and a hepatocellular cancer model, ethanol extract from *Citrus reticulata* (*C. reticulata*) peels was found to decrease proliferation by activation of p53 expressions in a dose-dependent fashion [46]. Furthermore, the action of tangeretin on the hallmarks of apoptosis has been reported, including B-cell lymphoma-2 gene (Bcl-2) [47, 48], caspases [32, 49], and DNA fragmentation [45, 47] in different cancer cell lines.

### 2.5. Effect on Angiogenesis and Metastasis

Cancer cell proliferation in addition to metastasis depends on angiogenesis for the adequate supply of oxygen and nutrients [10]. Several proteins have been recognized as angiogenic activators, including bFGF, TGF-*α*, TGF-*β*, TNF-*α*, IL-8, PDGF, G-CSF, PGF, HGF, and vascular endothelial growth factor (VEGF). In neoplastic vascularization, VEGF is considered a main mediator and has an important influence on cancer progression [50]. Some angiogenic proteins can be evoked by hypoxia [51] which triggers the expression of VEGF and its receptor through hypoxia-inducible factor-1*α* (HIF-1*α*) [52]. In addition, in silico studies reported the effect of tangeretin on ERK-2 and HIF1- *α* [10]. Tangeretin has been shown to act on the expression of VEGF to control certain pathological conditions such as tumor angiogenesis and metastasis in human ovarian [53] and lung [47] cancer cells. Further exploration into the mechanisms of TG may reveal promising insights into its underlying anticancer mechanism inducing angiogenesis.

### 2.6. Estrogenic Effect of Tangeretin

Estrogen has a critical contribution to the growth and differentiation of mammary and uterine cells and, therefore, might provoke cancer development [54]. Generally, estrogen has additional important roles in maintaining lipid level, genital tissue, and bone density [55]. The role of estrogen in preserving bone density is achieved by increasing bone formation and inhibiting the process of bone resorption. Binding estrogen to estrogen receptor (ER) activates or inhibits the copying of specific genes that respond to estrogen. In addition, the complex formation of estrogen-ER might show certain interaction with factors responsible for transcription such as specificity protein 1 (SP-1) [56]. Therefore, targeting the estrogenic receptor may open the door to chemoprevention and a cancer-oriented therapy [57]. Molecules with estrogen-like effects are known to have estrogenic effects, and many phytochemical compounds have been shown to possess certain activities such as phytoestrogens. The association between estrogen and ER may also activate some transcription factors such as c-Myc which later replicates genes and induces cell cycle activation. Treatment with *Citrus reticulata* was reported to inhibit the proliferation of MCF-7 when combined with doxorubicin, to promote the growth of rat mammary glands and increase rat uterus volume by inducing c-Myc production [10]. Tangeretin has been reported to increase the expression of the powerful invasion suppressor, E-cadherin/catenin [58], which is upregulated by estrogen in breast cancer cells [59]. This finding was confirmed by the ability of tangeretin to inhibit DMBA-induced breast cancer through the modulation of the expression of expressed estrogen and progesterone receptors [33]. Furthermore, the role of tangeretin, to restore bone loss provoked by estrogen, was reported previously in a mixture with other PMF [60].

### 2.7. Tangeretin Inducing Autophagy Process

Autophagy is a vesicle and lysosome-mediated process that is responsible for removing cytoplasmic waste from the cell and recycling some of its components to maintain hemostasis [61]. In cancer, the autophagic pathway plays a dual role in tumor promotion and suppression. It is believed that basal autophagy is a factor in cancer suppression because of the inability to dilute aberrant components through cell division. Reduced and abnormal autophagy inhibits the degradation of damaged components or proteins in oxidative-stressed cells, leading to cancer growth. Tangeretin derivative 5-acetyloxy-6, 7, 8, 4′-tetramethoxyflavone (5-AcTMF) was reported to induce autophagy in CL1-5 non-small cell lung cancer (NSCLC) by inducing the synthesis of autophagosome as well as increasing the expression of microtubule-associated proteins 1A/1B light chain 3B (LC3-II) and LC3 [62]. These data suggest that tangeretin enhanced autophagy is a key mechanism in the suppression of tumor generation.

### 2.8. Tangeretin in Inflammation

Chronic inflammation is associated with the onset or progression of many diseases including cancer. Many signaling pathways of inflammation are involved. Inflammatory markers such as interleukin-2 (IL-2), interleukin-6 (IL-6), tumor necrosis factor alpha (TNF-*α*), nuclear factor kappa-B (NF-*κ*B), and chemokines further enhance pathogenic processes [63]. Polymethoxyflavones were reported to prevent the expression and synthesis of TNF-*α* and other proinflammatory cytokines. [64]. The anti-inflammatory effect of tangeretin has been reported in different pathological conditions such as allergic rhinitis via stimulating regulatory T cell [65], asthma by regulating phosphoinositide 3-kinase (PI3K) pathway [21], and Respiratory Syncytial Virus-induced inflammation by curbing NF-*κ*B and interleukin-1 beta (IL-1B) [23]. Furthermore, tangeretin alleviated cisplatin-induced renal inflammation via inhibiting NF-*κ*B activation and decreasing its downstream IL-1B and TNF-*α* [66], as well as iNOS and TNF-*α* during reinstatement of the anti-inflammatory interleukin-10 (IL-10) [67]. In addition, we have reported previously the ability of tangeretin to decrease cisplatin-induced hepatic inflammation by decreasing TNF-*α* and boosting IL-10 [17].

## 3. Absorption and Oral Bioavailability of Tangeretin

Tangeretin has a significant advantage over other chemically relevant flavones as it shows large intestinal absorption and therefore is bioavailable [68]. It is also considered safe when administered orally [69]. The TG kinetics was tested by collecting Hamster's urine and plasma after 35 days of free access to food containing 1% tangeretin. Intestinal absorption of tangeretin was noticeable with respect to the excretion of several metabolites in urine. Animal plasma was almost free of any unchanged tangeretin [68]. Nevertheless, some biologically active botanical chemicals have low bioavailability and poor solubility. In these chemicals, the process of adding an acetyl group to the existing substance is usually used to get a drug derivative that helps improve the uptake and effectiveness of targeted natural molecules. For this reason, the derivative of tangeretin, 5-AcTMF, has been used in numerous studies [62].

## 4. Safety and Toxicity of Tangeretin

In order to investigate the possibility of oral toxicity, tangeretin was used as a typical compound for safety assessment as it is considered one of the most common PMF that originated from natural sources [70]. In a study carried out by Ting et al. to examine the acute oral toxicity in mice genders, tangeretin was administrated in 1000, 2000, and 3000 mg/kg from a single gavage in an oil suspension. Outcomes were no deaths observed 14 days after administration. However, daily use of low dose tangeretin displayed a U-shaped dose-response curve including hepatic changes. It was concluded that PMF available as a beneficial ingredient in the human diet could be applied safely at different conditions [70]. Consistent with the previous study, Vanhoecke et al. proved the safety of Tangeretin when administrated orally to experimental mice. Evidence included the absence of any major harm to body organs or deterioration in function. These results open the door for additional safety evaluations in humans [69].

Possible genotoxicity of tangeretin has been investigated by Delaney et al., who administered a PMF mixture in vitro using a range of concentrations in five different bacterial strains. Results indicated no mutations detected regardless of ribosomal protein S9 activation. Observed results indicate the safety profile of PMF mixture and excluded any possibility of genotoxicity from in vitro assay systems [71]. However, in another study, Delaney et al. showed a statistically insignificant positive relationship between increasing the concentrations of PMF and spleen weight in sheep red blood cell (SRBC) immunized mice. In mice without immunization, there was no evidence for spleen weight changes [64].

## 5. Tangeretin-Drug Interactions

In the last two decades, many studies discussed citrus fruit-drug interactions. The simultaneous intake of tangeretin and drugs was reported to have a strong influence on pharmacokinetics. Citrus juice was demonstrated to affect the pharmacokinetics of various kinds of drugs by modulating drug transporters and drug-metabolizing enzymes [72]. For instance, the flavonoid fraction of clementine juice can induce or inhibit a number of human cytochrome enzymes such as CYP3A4 and CYP1A2 [73]. Mandarin juice inhibited CYP3A4, resulting in increased concentration of tacrolimus in patients [72]. The mean felodipine plasma concentration-time area under the curve (AUC) showed to be elevated due to the inhibition of CYP3A4 activity after lime juice consumption [74].

Tangeretin showed significant inhibition on P-gp in (multidrug resistance protein 1) MDR1-MDCKII cells, which might be the reason underlying the increased cell toxicity of paraquat and taxol. In addition, concurrent administration of digoxin with tangeretin has been reported to alter the pharmacokinetics of digoxin [75].

Tangeretin is present in the bitter orange peel of Seville orange, the Seville juice was reported to inhibit intestinal cytochrome P450 (CYP) isozyme 3A4, and P-glycoprotein was shown to affect colchicine metabolism and transport, which can lead to severe toxicity [76]. Sildenafil bioavailability is reported to be improved by Seville orange; this can be contributed to the inhibition of its intestinal first-pass effect of CYP3A4 and/or P-gp [77]. On the other hand, tangeretin was reported as a potent regioselective stimulator causing CYP3A4 induction [73, 78]. This stimulation can alter midazolam metabolism [78]. Thus, flavonoid food contents should be reported to avoid any unpredictable scenarios.

These studies highlight the need for further investigations to confirm the correlation between the *in vitro* and *in vivo* results concerning tangeretin-drug interaction.

## 6. Tangeretin Application

The effect of tangeretin on different cancer types is summarized in Table 1 and detailed below.

### 6.1. Ovarian Cancer

Ovarian cancer is considered the second most fatal cancer among females in developed regions [99]. Ovarian cancer is difficult to cure because of the resistance that arises towards chemotherapy. Consequently, it was important to identify new and effective chemotherapeutic agents [53]. Despite many women who show a good response to first-line therapy in ovarian cancer, disease recurrence is very common due to resistance to chemotherapeutic agents. Resistance to chemotherapeutic agents in turn is a prime hindrance to improving the diagnosis of ovarian cancer. Subsequently, it is deemed necessary for research regarding ovarian cancer to seek new chemical treatment agents from natural sources [53].

A study conducted by He et al. assessed the impact of tangeretin on the articulation of VEGF and cell proliferation in two different cell lines of ovarian cancer [53]. They reported a modest suppressing effect on cell proliferation for OVCAR-3 and A2780/CP70 cells. Furthermore, tangeretin demonstrated some inhibitory effects on VEGF expression at the OVCAR-3 and A2780/CP-70 cell line [53].

Moreover, the vast majority of ovarian cancer patients are not perfectly treated with the standard therapy of cisplatin [cis-diamminedichloroplatinum(II)] primarily because of the impediment developed with drug resistance [100]. However, when using flavonoids alone, it was able to induce cell death for certain cancer cells while regenerating normal cells [101]. In our study, the potentiality of tangeretin to sensitize resistant ovarian cancer cells to cisplatin was examined and its effect to induce apoptosis was confirmed [31].

### 6.2. Gastric Cancer

Gastric cancer is considered the second main reason for death associated with cancer over the world [102]. Adenocarcinoma gastric cell line (AGS) is a kind of human gastric mucous cell carcinoma with wild-type p53, which has been used in many studies of antitumor drugs [103]. However, in some cancerous cells, mutation of p53 might lead to p53 inactivation and lose its tumor-suppressive activity [104].

Dong et al. illustrated that AGS when treated with dose-dependent tangeretin, a reduction in the mitochondrial membrane potential (MMP) is shown. A significant manifestation in apoptosis caused by tangeretin is mitochondrial dysfunction [32]. Upregulation of bcl-2-like protein 4 (Bax) activates p53 to induce apoptosis mediated by mitochondria which will contribute to activation of caspase-9 and consequently the downstream caspases in this pathway. Moreover, pifithrin-*α* (PFT-*α*), p53 inhibitor, will suppress the expression of p53, p21, caspase-3, and caspase-9, thus, the apoptotic effect that is mediated by tangeretin. In conclusion, data indicated that tangeretin stimulated programmed cell death of AGS cells primarily through dysfunction of mitochondria dependent on p53 as well as external pathways mediated by Fas/FasL [32].

### 6.3. Lung Cancer

Lung cancer is identified as the main cause of cancer-related mortality in the world. It is classified into three main categories: lung carcinoid tumor, small cell lung cancers (SCLCs), and NSCLCs. The most common lung cancer type is NSCLC which constitutes almost 85% of cases. Concerning the treatment of NSCLC patients, chemotherapy and radiotherapy results are weak due to drug resistance development and the mechanism of cell protection [62].

However, the effect of 5-AcTMF as an anticancer agent on CL1-5 of NSCLC has been examined both *in vivo* and in the laboratory. Several actions for 5-AcTMF were noticeable, such as suppression of tumor proliferation, arresting G2/M checkpoint that is linked with cell division control 2 (CDC2), and cell division cycle 25C (CDC25C). In addition, there was an elevation in the number of apoptotic cells, activation of caspases pathway, decrease in Bcl-2, XIAP, and survivin expression encouraging the liberation of cytochrome C inside the cytosol and MMP disturbances. Moreover, a curbing effect of 5-AcTMF has been observed on the PI3K/protein kinase B/mammalian target rapamycin (PI3K/Akt/mTOR) as a signaling pathway. Akt is expressed excessively by Akt-cDNA transfection as it is inhibited through 5-AcTMF-mediated programmed cell death and autophagocytosis advocating the activation of apoptosis by suppressing the pathway of Akt. Experimentally, it has been found that the growth of the CL1-5 cell line was delayed in such a manner as to achieve the desired results by 5-acTMF therapy, with no proof of deleterious effects [62].

In another study, Chen et al. investigated the effects of tangeretin on COX-2 expression levels in H1299, a human NSCLC and A549, and lung epithelial carcinoma cells. They found that when the cell is pretreated with tangeretin that inhibition of certain signaling factors is induced by IL-1B. Signaling factors include p38 mitogen-activated protein kinases (p38 MAPKs), c-Jun N-terminal kinase (JNK), and AKT phosphorylation excluding deactivation of downstream NF-*κ*B. The above observations disclose that, with an A549 cell type, the inhibition of tangeretin to IL-1B-induced COX-2 expression is accomplished through deactivation of the NF-*κ*B transcription controller in addition to hindering the process of signaling factors, while not including extracellular signal-regulated kinases [79].

### 6.4. Prostate Cancer

Prostate cancer is classified in the US as the second common cancer type as well as the second major cause of death in the western world. Almost 25% of male patients diagnosed with cancer in the US of America are confirmed as having prostate cancer. However, the underlying causes of prostate cancer remain unclear as there has been no particular cancer-causing substance discovered [88].

Zhu et al. assessed tangeretin's effect on two different types of prostate cancer, androgen-insensitive prostate cancer cells (PC-3) and androgen-sensitive human prostate adenocarcinoma cells (LNCaP). Tangeretin showed dosage- and time-dependent inhibition, where the 50% inhibitory concentration (IC50) after 72 hours was noted at 75 *μ*M and 65 *μ*M in both cell lines, respectively [80]. Results of tangeretin treatment in PC-3 clearly exhibited a reduction in mesenchymal proteins containing vimentin, cluster of differentiation 44 (CD44), and cadherin-2 (CDH2). Upregulation was observed with epithelial proteins containing cadherin-1 and cytokeratin-19. An additional result was the suppression of the PI3K/Akt/mTOR pathway. Consequently, it was demonstrated that tangeretin in PC-3 cells stimulated reprogramming of epithelial–mesenchymal transition (EMT) through directing the PI3K/Akt/mTOR pathway to act as the fundamental mechanism of action for inducing toxicity. Therefore, tangeretin offers an unfamiliar approach for prostate cancer treatment [80].

### 6.5. Leukemia

Tangeretin was observed by Ishii et al. to have an inhibitory effect on cell proliferation, function of P-gp, and significantly affected the cell cycle of human acute T lymphoblastic leukemia (MOLT-4). In addition, tangeretin had an inhibitory effect on cells which exhibit resistance to daunorubicin, a chemotherapeutic agent. However, tangeretin does not stimulate apoptosis [81]. Similarly, the effect of tangeretin on the uptake of [(3)H]vincristine into DOX-resistant human myelogenous leukemia cells (K562/ADM) was tested by Ikegawa et al. Their study found that, by inhibiting the efflux mediated by P-gp for [(3)H]vincristine, that accumulation of chemotherapy drugs occurred within the cells [82]. In contrast to Ishii et al., tangeretin has shown to promote apoptosis in HL-60 cells through DNA fragmentation and reduction of G1 cells along with an increase in the S and/or G2/M cells without any evidence of toxicity towards human peripheral blood mononuclear cells (PBMCs) [45].

The antitumor effect of tangeretin was studied on murine leukemia type P388 in a living organism. The results of that extract proved tangeretin activity in both *in vivo* and the laboratory. Accordingly, tangeretin showed an inhibitory effect on cell growth in both leukemia L1210 and K562 cell lines [83]. Also, Mak et al. showed the effects of tangeretin on the growth and differentiation of a newly recognized murine myeloid leukemia cell line (WEHI-3B JCS). Both *in vitro* and *in vivo* proliferation of JCS leukemic cells which were treated with tangeretin were critically curtailed. However, the rate of survival of rats with JCS tumor cells receiving tangeretin increased [84].

### 6.6. Melanoma

Melanoma is one of the prevailing malignant tumors *s* characterized by metastasis. A study done by Martínez et al. showed that Swiss mice that received flavonoid treatment developed suppression for metastasis when compared to an ethanol group at the same index [105]. In another experiment, it was found that treatment with tangeretin 25 *μ*M in B16/F10 murine skin cancer cells catalyzed the production of melanin within cells through activation of melanogenic protein expressions such as tyrosinase, tyrosinase-related protein (TRP)-1, and ERK 1/2. Moreover, CREB and MITF expression was higher in one hour and four hours, respectively. Studies have shown a curative power for tangeretin in skin cancer and the associated depigmentation [85]. In addition, the effect of tangeretin was examined by Yoon et al. using mouse skin epidermal JB6P + cells to prove an inhibitory effect of tangeretin on COX-2 expression as well as the transactivation of NF-*κ*B and activator protein 1. This was accomplished by inhibiting phosphorylation of Akt and MAPKs which include JNK, ERK, p38, and decreased the phosphorylation of MAPK kinases 1/2, 3/6, and 4. In addition, the capability of internal generation ROS was minimized by tangeretin, thus preventing further oxidative stress for healthy cells [86].

According to Rodriguez et al., SK-MEL-1 and B16F10 skin cancer cell lines responded favorably to tangeretin. They indicated that hydroxylated flavonoids with absent double bond C2–C3 lead to loss of effectiveness on both melanoma cell lines. However, tangeretin showed the highest efficacy and this is due to the availability of a minimum of 3 methoxyl groups which provides a more effective antiproliferative effect [87]. Similarly, tangeretin's effects have been studied by Kandaswami et al. in the growth of a human squamous cell carcinoma cell line (HTB43) and have shown that significant cell growth suppression can be attributed to a higher membrane uptake [88,89].

### 6.7. Brain Cancer

Recurrent meningioma is a rare but serious problem occurring after the failure of standard treatment (surgery and radiation). The current chemotherapies have been considered as regimens with only a slight benefit. Thus, there is an urgent need for effective treatments for meningioma patients who have tried standard therapies but without useful results [90].

Das et al. provided powerful preliminary evidence for the curative effect of tangeretin in IOMM-Lee and CH157MN meningioma cells. They found that tangeretin acts by inducing cell death with phosphorylation of glycogen synthase kinase 3 *β* (GSK3*β*) through the suppression of Wnt5/*β*-catenin pathway. In addition to apoptosis, tangeretin stimulated downregulation processing of the tetraspanin protein (TSPAN12) and survival proteins (Mcl-1 and Bcl-XL), while upregulating apoptotic factors (Bax and caspase-3) [90]. Ma et al. reported similar results for tangeretin-treated U-87 MG and LN-18 cells, as they markedly demonstrated cell growth inhibition and apoptotic effects when compared to nontreated cells. It has been reported that tangeretin acts by the mechanism of modifying phosphatase and tensin homolog (PTEN) together with genes responsible for cell cycle regulation such as cyclin-D, cdc2 mRNA, and protein expressions [91]. However, a study reported by Rooprai et al. shows the effect of tangeretin on different criteria of brain tumor invasion such as expression of matrix metalloproteinase migration, adhesion, and invasion revealing that tangeretin demonstrated a significant downregulation effect of MMP-2 and MMP-9 in the grade 3 astrocytoma. Moreover, in several cell lines such, as anaplastic astrocytoma, ependymoma-a grade II oligoastrocytoma, and glioblastoma multiform, citrus flavonoids showed great inhibition of invasion, migration, and adhesion [92].

### 6.8. Breast Cancer

At a global level, breast cancer is increasingly alarming as it is the second most common cancer in females. Genetic factors are attributed to only 10% of cases reported with breast cancer, while the most prevalent causes are environmental including diet, which constitutes the most important role in breast cancer prevention [33].

Arivazhagan and Pillai reported that tangeretin can greatly slow antitumor activity through an inhibitory effect on estrogen, progesterone, and prolactin serum level, as well as lipid bound sialic acid (LBSA), total sialic acid (TSA), and levels of nitric oxide and protein carbonyls in tissues of animals with DMBA-induced breast cancer. Furthermore, tangeretin oral treatment decreased signs of tumor cells such as proliferating cell nuclear antigen (PCNA), COX-2, and Ki-67 and affected cell division by upregulating p53/p21 and secondary suppression of metastasis by inhibiting MMP-2, MMP-9, and VEGF [93]. Similarly, it was found that tangeretin therapy in human MCF-7/6 breast cancer cells showed a great anti-invasive as well as antiproliferative effect when tangeretin was applied *in vitro*. Moreover, a reduction in NK cells was observed [94]. Consistent with the previous studies, tangeretin-treated MDA-MB-468, MDA-MB-435, and MCF-7 cells showed an antiproliferative effect attributed to arresting the cell cycle in G1 phase [12, 42], as well as activation of CYP1 and expression of CYP1A1/CYP1B1 that document the ability of tangeretin to prevent the spread of breast cancer cells by the metabolism-mediated processes through CYP1A1/CYP1B1 and 4′hydroxy tangeretin in both MCF-7 and MDA-MB-468 [12].

Abe et al. pointed out that tangeretin when administered to the mammary gland of a mouse with an induced tumor demonstrated inhibition of atypical hyperplastic lesion and stimulated the programmed death of ductal epithelial cells [106]. However, Morley et al. (2007) disagreed with the ability of tangeretin to procure apoptosis in both MDA-MB-435 and MCF-7 breast cancer cell lines. Rather, they indicated that tangeretin is a cytostatic agent causing inhibition of proliferation with no evidence of programmed cell death [42]. The effectiveness of tangeretin was clearly demonstrated by two studies as a potent suppressor of breast cancer in rats induced by DMBA. Data showed higher performance of the serum enzymes such as liver function biomarkers, alkaline and acid phosphatases, *γ*-glutamyltransferase (*γ*-GT), 5′-nucleotidase (5′-ND), and lactate dehydrogenase (LDH) in rats with breast cancer, reduced to levels close to normal by the administration of tangeretin. Moreover, some enzymatic and nonenzymatic antioxidants and thiobarbituric acid reactive substances (TBARS), a byproduct of lipid peroxidation, along with both phases of detoxification showed a significant reduction as a result of tangeretin treatment [29,33]. Lakshmi and Subramanian added to the inhibitory effect of tangeretin in some oxidative stress markers and reported that tangeretin also significantly improved the level of endogenous antioxidants in kidney tissue. This result demonstrates the expression of nuclear factor (erythroid-derived 2)-like 2/Kelch-like ECH-associated protein 1 (Nrf2/Keap1) in renal tissues within the normal range, hence, protecting kidneys efficiently from oxidative damage by DMBA and confirming tangeretin's nature as a nephroprotective agent [36]. Periyasamy et al. demonstrated that tangeretin plays a specific role in regulating the flow of cellular metabolic energy in DMBA-induced breast cancer-bearing rats. However, treated rats with tangeretin exhibited normalization in the level of glycolytic enzymes as well as a significant rise in the activities of the citric acid cycle and respiratory chain enzyme. Moreover, the expression of PCNA was downregulated [95].

### 6.9. Liver Cancer

A study reported by Kurowska et al. revealed a significant reduction in the secretion of apolipoprotein B (apoB) and suppression of cholesteryl esters, free cholesterol, and triacylglycerol (TAG) intracellular synthesis upon incubation with tangeretin in human hepatoma cell line HepG2. Cellular triacylglycerol was also decreased in size. These results were correlated with the reduction in microsomal triglyceride transfer protein (MTTP) and diacylglycerol acyltransferase (DGAT) activities. Moreover, tangeretin showed activation of the transcription factor, peroxisome proliferator-activated receptor (PPAR), which is responsible for controlling the oxidation process of fatty acids and triacylglycerol in a positive manner [107].

However, in another study, tangeretin exhibited antagonistic action against the inhibition of gap junctional intercellular communication (GJIC) caused by tumor stimulators such as 3,5,di-tertio-butyl-4-hydroxytoluene (BHT) and 12-O-tetradecanoyl-phorbol-acetate (TPA) in rat liver epithelial cell line (REL) [96, 97].

### 6.10. Colorectal Cancer

Colorectal cancer (CRC) is one of the leading causes of cancer death in adults and has been linked to many lifestyle-related factors [108]. Silva et al. identified tangeretin as an agent that prevented the spread of colorectal cancer through a different mechanism of action on spheroid cells of HT29 colon cancer cell line. This mechanism includes the antiproliferation effect, disruption of cell cycle (G2/M phase), inhibition of aldehyde dehydrogenase (ALDH+), and a cancer stem cell marker and inducing apoptosis [98]. Similar results have been reported by Fan et al. on intestinal tumor growth of a mouse model for human familial adenomatous polyposis (FAP) that demonstrated further increased apoptosis of intestinal tumors after been fed 0.5% of orange peel extract that is rich in tangeretin [109]. More specifically, Pan et al. illustrated that the antiproliferative effect of tangeretin in COLO 205 was achieved by either modifying the expression of many regulatory proteins that are major for G1 phase, like CDK2 and CDK4, or stimulating the activities of both p21 and p27, cyclin-dependent kinase inhibitors [30].

## 7. Tangeretin as a Potential Adjuvant Chemotherapeutic Agent

Among available treatment options for cancer, chemotherapy is the most effective therapy for treating a variety of cancers. Chemotherapeutics are mainly classified based on their mechanism of action and their chemical structure. Unfortunately, chemotherapy exhibits undesirable side effects which can lead to dose reduction or even cessation of treatment. Combined chemotherapy may increase the effectiveness of therapeutic chemical agents; this, in turn, permits the use of lower doses of the chemotherapeutic agent and hence reduces toxicity to normal tissues [110, 111]. Increased efficacy can be achieved by combining agents possessing a chemotherapeutic effect that has an additional or synergistic effect, whereas toxicity can be reduced by using agents that have an immunomodulatory effect [112].

Interestingly, many studies have validated the anticancer properties of tangeretin. Bracke et al. in 1999 reported that tangeretin reversed the antiproliferative effect of tamoxifen on tumor cells in human MCF-7/6 mammary adenocarcinoma cells induced in female nude mice [113]. On the other hand, studies on citrus flavonoids have shown that when combined with chemotherapy, tangeretin has significantly increased drug anticancer efficacy on resistant cancer cell lines. Concurrent use of tangeretin with chemotherapeutic agents synergistically stimulated cell death and cell cycle arrest in resistant cancer cells [31].

Published data showed that tangeretin has the ability to sensitize excessive ABCB1 expression cancer cells to chemotherapeutic agents through direct inhibition of ABCB1 transporter activity. This in turn motivated further studies in animals as well as clinical trials for the purpose of overcoming cancer resistance [114]. In a study done by Akao et al. combining tangeretin with 5-demethyl, nobiletin caused cell apoptosis by limiting MMP and raising the assumption that, through the stated combination therapy, an intrinsic process of programmed cell death will be activated by a synergetic effect. These findings suggested the importance of phytochemical combinations for enhancing the cancer-preventive effect [49]. We have reported previously that combination treatments of cisplatin and tangeretin on A2780/CP70 and 2008/C13 cisplatin-resistant human ovarian cancer cells downregulated PI3K/Akt pathway and consequently curbed NF-*κ*B and BAD making resistant cells more prone to the toxic effects of cisplatin. Additionally, the downregulation of GSK-3*β* accounts for the synergetic effect of cisplatin and tangeretin combination on apoptosis. In line with flow cytometry analysis, this combination treatment also reduced cdc25c gene and cyclin B1 protein levels while p53 was significantly increased. Overall, cisplatin-tangeretin combination therapy provides a novel approach for patients with ovarian cancer and provides an effective treatment regimen [31]. Recent findings from our laboratory indicate that tangeretin has the ability to protect against cisplatin-induced liver and kidney toxicities. This protection was facilitated by modulating different inflammatory mediators, apoptotic, oxidative, and survival signaling pathways [17, 67].

## 8. Conclusion

In conclusion, it is evident that tangeretin, an extract from citrus peels, has a wide range of positive effects. In our review, we focused on tangeretin as a therapeutic approach in different cancer cell lines with various properties including antioxidant, activating apoptosis, arresting cell cycle, antiangiogenesis, and antiproliferative, which are supported abundantly with evidence-based research detailing the mechanism of action. In addition, using tangeretin in combination with chemotherapeutic agents may be considered as an option for enhancing the efficacy of these agents. Favorable effects of tangeretin presented in this paper encourage the use of this natural agent as a drug with a broad spectrum of medical applications. Further and comprehensive clinical research is needed to prove its beneficial role and effectiveness as a pharmaceutical preparation.

## Figures and Tables

**Table 1:** Summary of the anticancer effects of tangeretin.

	Cell line	Function	Proposed mechanism of action	Reference
Ovarian cancer	OVCAR-3	Antiangiogenesis	Decrease in VEGF expression.	He et al. [53]
	A2780/CP70			
	A2780/CP70	Cell cycle regulator	Caspase cascade, cell cycle arrest at G(2)-M phase.	Arafa et al. [31]
	2008/C13	Apoptosis inducer	Downregulation of phospho-Akt along with NF-KB, phospho-GSK-3beta, and phospho-BAD.	

Gastric cancer	AGS	Apoptosis inducer	Reduction in the MMP.	Dong et al. [32]
			Upregulation of BAX with p53 activation.	

Lung cancer	CL1-5	Apoptosis inducer	Decreasing expression of Bcl-2, XIAP, and survivin.	Li et al. [16]
			Disturbance in MMP.	
			Translocation of cytochrome C to the cytosol.	
	H1299	Antiproliferative	Inhibition of IL-1B-induced COX-2 expression.	Chen et al. [79]
	A549	Apoptosis inducer	Deactivation of NF-*κ*B downstream.	

Prostate cancer	PC-3	Antimetastatic	Downregulation of mesenchymal proteins.	Zhu et al. [80]
			Upregulation of epithelial proteins.	
		Antiproliferative	Downregulation of PI3K/Akt/mTOR pathway.	

Leukemia	MOLT-4	Reverse MDR	Inhibition of P-glycoprotein function.	Ishii et al. [81]
	K562/ADM	Reverse MDR	Inhibition of P-glycoprotein-mediated efflux of [(3)H]vincristine.	Ikegawa et al. [82]
	HL-60	Apoptosis inducer	Lowering DNA content.	Hirano et al. [45]
		Cell cycle regulator	Decrease of G1 cells, increase of S and/or G2/M cells.	
	L1210	Antiproliferative	Inhibition of cell growth	Satoh et al. [83]
	K562			
	JCS	Autophagy inducer	Increase in phagocytic activity of the cells.	Mak et al. [84]

Melanoma	B16F10	Melanogenesis	Increasing levels of tyrosinase, TRP-1, and ERK 1/2	Yoon et al. [85]
			Increasing expression of CREB and MITF	
	JB6 P+	Anti-inflammatory	Blockage of Akt, MAPKs, and MAPK kinases.	Yoon et al. [86]
		Antioxidant	Scavenging ROS.	
	B16F10	Antiproliferative	Inhibition of cell growth.	Rodriguez et al. [87]
	SK-MEL-1			
	HTB43	Antiproliferative	Greater membrane uptake.	Kandaswami et al. [88]
				Kandaswami et al. [89]

Brain cancer	IOMM-Lee	Apoptosis inducer	Enhancing phosphorylation of GSK3*β*.	Das et al. [90]
	CH157 MN		Downregulation of TSPAN12, Bcl-XL, and Mcl-1.	
			Overexpression of Bax and caspase-3.	
	U-87MG	Cell cycle regulator	Increases G2/M arrest.	Ma et al. [91]
	LN-18	Apoptosis inducer	Modulating PTEN and cell-cycle regulated genes (cyclin-D and cdc-2 mRNA and protein expressions).	
	Grade III astrocytoma	Antimetastatic	Downregulation of MMP-2 and MMP-9	Rooprai et al. [92]

Breast cancer	DMBA-induced rat mammary carcinoma	Antitumor	Decrease in levels of estradiol, progesterone, and prolactin.	Arivazhagan and Pillai [93]
		Antioxidant	Decrease in levels of LPSA, TSA, NO, and protein carbonyls.	
		Antiproliferative	Reduction in PCNA, COX-2, and Ki-67 markers.	
		Cell cycle regulator	Arresting cell division at the G1/S phase via p53/p21 upregulation.	
		Antimetastatic	Downregulation of MMP-2, MMP-9, and VEGF.	
	MCF-7/6	Immune-modulator	Reducing the number of NK cells.	Depypere et al. [94]
	MCF-7 MDA-MB-435	Antiproliferative	Blocking cell cycle progression at G1.	Morley et al. [42]
	MCF7 MDA-MB-468	Antiproliferative	Blocking cell cycle progression at G1.	Surichan et al. [12]
			CYP1A1/CYP1B1-mediated metabolism to 4′ hydroxy tangeretin.	
	DMBA-induced mammary carcinoma	Antioxidant	Normalizing levels of AST, ALT, ALP, ACP, 5′-ND, *γ*-GT, and LDH.	Lakshmi and Subramanian [36]
			Decrease in TBARS, enzymatic, nonenzymatic antioxidants, phase I and phase II detoxification.	Periyasamy et al. [29]
	DMBA-induced mammary carcinoma	Antioxidant	Normalizing activities of glycolytic enzymes.	Periyasamy et al. [95]
			Increasing activities of citric acid cycle and respiratory chain enzyme.	
		Antiproliferative	Downregulation of PCNA expression.	

Liver cancer	HepG2	Antioxidant	Suppressing of TAG synthesis and mass.	Kurowska et al. [68]
			Decreasing activities of DAG acyltransferase and microsomal triglyceride transfer protein.	
			Activating PPAR.	
	REL	Antiproliferative	Upregulation of GJIC.	Chaumontet et al. [96]
				Chaumontet et al. [97]

Colorectal cancer	HT29	Cell cycle regulator	Arresting G2/M phase with reduction in ALDH+.	Silva et.al. [98]
	COLO 205	Cell cycle regulator	Blocking cell cycle progression at G1 phase.	Pan et al. [30]
		Antiproliferative	Inhibiting the activities of Cdk2 and Cdk4.	
		Apoptosis inducer	Increasing in p21, p27, and p53 levels.	

## Data Availability

No data were used to support this study. It is a review article.

## Abbreviations

5-ACTMF: 5-Acetyloxy-6, 7, 8, 4′-tetramethoxyflavone
5′-ND: 5′-Nucleotidase
8-OHdG: 8-Hydroxy-2′-deoxyguanosine
AGS: Adenocarcinoma gastric cell line
ALDH+: Aldehyde dehydrogenase
AUC: Area under the curve
apoB: Apolipoprotein B
Bax: bcl-2-like protein 4
Bcl-2: B-cell lymphoma-2 gene
Bcl-XL: B-cell lymphoma-extra large
bFGF: Basic fibroblast growth factor
BHT: 3, 5,di-Tertio-butyl-4-hydroxytoluene
CD44: Cluster of differentiation 44
CDC2: Cell division control 2
CDC25 C: Cell division cycle 25 C
CDH2: Cadherin-2
CDK: Cyclin-dependent kinases
CDK2: Cyclin-dependent kinase 2
CDK4: Cyclin-dependent kinase 4
c-JNK: c-Jun N-terminal kinase
CL1-5: Human lung cancer cell line
COLO 205: Human colon cancer
COX-2: Cyclooxygenase-2
CRC: Colorectal cancer
CREB: Cyclic adenosine monophosphate response element binding protein
DGAT: Diacylglycerol acyltransferase
DMBA: 7, 12-Dimethylbenz[a]anthracene
DOX: Doxorubicin
EGF: Epidermal growth factor
EMT: Epithelial-mesenchymal transition
ER: Estrogenic receptor
ERK-2: Extracellular signal-regulated kinase 2
G-CSF: Granulocyte colony-stimulating factor
GJIC: Gap junctional intercellular communication
GSK3*β*: Glycogen synthase kinase 3 *β*
HepG2: Human liver cancer cell line
HGF: Hepatocyte growth factor
HIF1-*α*: Hypoxia-inducible factor 1-alpha
HL-60: Human promyelocytic leukemia
HNE: 4-Hydroxy-2-trans-nonenal
HTB43: Human squamous cell carcinoma cell line
IC50: 50% inhibitory concentration
IFN-*γ*: Interferon gamma
IL: Interleukin
iNOS: Inducible nitric oxide synthase
LBSA: Lipid bound sialic acid
LC3: Light chain 3
LDH: Lactate dehydrogenase
LNCaP: Androgen-sensitive human prostate adenocarcinoma cells
MCF-7: Breast cancer cell line
Mcl-1: Induced myeloid leukemia cell differentiation protein
MITF: Microphthalmia transcription factor
MMP: Mitochondrial membrane potential
MMPs: Matrix metalloproteinases
MMP-2: Matrix metalloproteinase-2
MMP-9: Matrix metalloproteinase-9
MOLT-4: Human acute T lymphoblastic leukemia
MTTP: Microsomal triglyceride transfer protein
NF-*κ*B: Nuclear factor kappa-light-chain-enhancer of activated B cells
NK: Natural killer
Nrf2/Keap1: Nuclear factor (erythroid-derived 2)-like 2/Kelch-like ECH-associated protein 1
NSCLC: Non-small cell lung cancer
P-gp: P-Glycoproteins
p38 MAPKs: p38 mitogen-activated protein kinases
PBMCs: Peripheral blood mononuclear cells
PC-3: Androgen-insensitive prostate cancer cells
PCNA: Proliferating cell nuclear antigen
PDGF: Platelet-derived growth factor
PFT-*α*: Pifithrin-*α*
PGF: Placental growth factor
PI3K/Akt/mTOR: Phosphatidylinositol 3-kinase/protein kinase B/ mammalian target of rapamycin
PMF: Polymethoxyflavone
PPAR: Peroxisome proliferator-activated receptor
PTEN: Phosphatase and tensin homolog
SCLCs: Small cell lung cancers
SP-1: Specificity protein 1
SRBC: Sheep red blood cells
TAG: Triacylglycerol
TBARS: Thiobarbituric acid reactive substances
TGF: Transforming growth factor
TH+: Tyrosine hydroxylase positive
TNF-*α*: Tumor necrosis factor alpha
TPA: 12-O-Tetradecanoyl-phorbol-acetate
TRP: Tyrosinase-related protein
TSA: Total sialic acid
TSPAN12: Tetraspanin protein
VEGF: Vascular endothelial growth factor
WEHI-3B: JCS murine myeloid leukemia cell line
XIAP: X-linked inhibitor of apoptosis protein
*γ*-GT: *γ*-Glutamyltransferase.
